# Neurovascular effects of cocaine: relevance to addiction

**DOI:** 10.3389/fphar.2024.1357422

**Published:** 2024-02-22

**Authors:** Kevin Clare, Kicheon Park, Yingtian Pan, Carl W. Lejuez, Nora D. Volkow, Congwu Du

**Affiliations:** ^1^ New York Medical College, Valhalla, NY, United States; ^2^ Department of Biomedical Engineering, Stony Brook University, Stony Brook, NY, United States; ^3^ Department of Psychology, Stony Brook University, Stony Brook, NY, United States; ^4^ National Institute on Alcohol Abuse and Alcoholism, Bethesda, MD, United States

**Keywords:** cocaine, neurovascular coupling (NVC), neuroimaging, cerebral blood flow (CBF), addiction

## Abstract

Cocaine is a highly addictive drug, and its use is associated with adverse medical consequences such as cerebrovascular accidents that result in debilitating neurological complications. Indeed, brain imaging studies have reported severe reductions in cerebral blood flow (CBF) in cocaine misusers when compared to the brains of healthy non-drug using controls. Such CBF deficits are likely to disrupt neuro-vascular interaction and contribute to changes in brain function. This review aims to provide an overview of cocaine-induced CBF changes and its implication to brain function and to cocaine addiction, including its effects on tissue metabolism and neuronal activity. Finally, we discuss implications for future research, including targeted pharmacological interventions and neuromodulation to limit cocaine use and mitigate the negative impacts.

## 1 Introduction

Cocaine disrupts brain function, including resting state activity, response to stimulation, and functional connectivity between brain regions. These functional impairments might result from (or be associated with) decreases in cerebral blood flow (CBF) observed in cocaine misusers and in laboratory animals with chronic cocaine exposures. Though the mechanisms underlying cocaine-induced CBF reductions are not fully understood they are likely to reflect: 1) direct vasoconstrictive effects of cocaine and 2) indirect effects from reduced neural activity and metabolic demand (Volkow et al., 1988). The severity, brain regional locations, and the mechanism underlying cocaine’s CBF reductions in turn will affect the characteristics of the brain dysfunction. Here we summarize our preclinical findings using *in-vivo* optical neuroimaging along with a literature review of preclinical and clinical studies. This review aims to provide an overview of cocaine-induced changes in the brain’s CBF and metabolism, which may result in brain dysfunction and contribute to neurotoxicity and cognitive deficits that perpetuate addictive behaviors.

## 2 Literature search strategy

**FIGURE 1:**
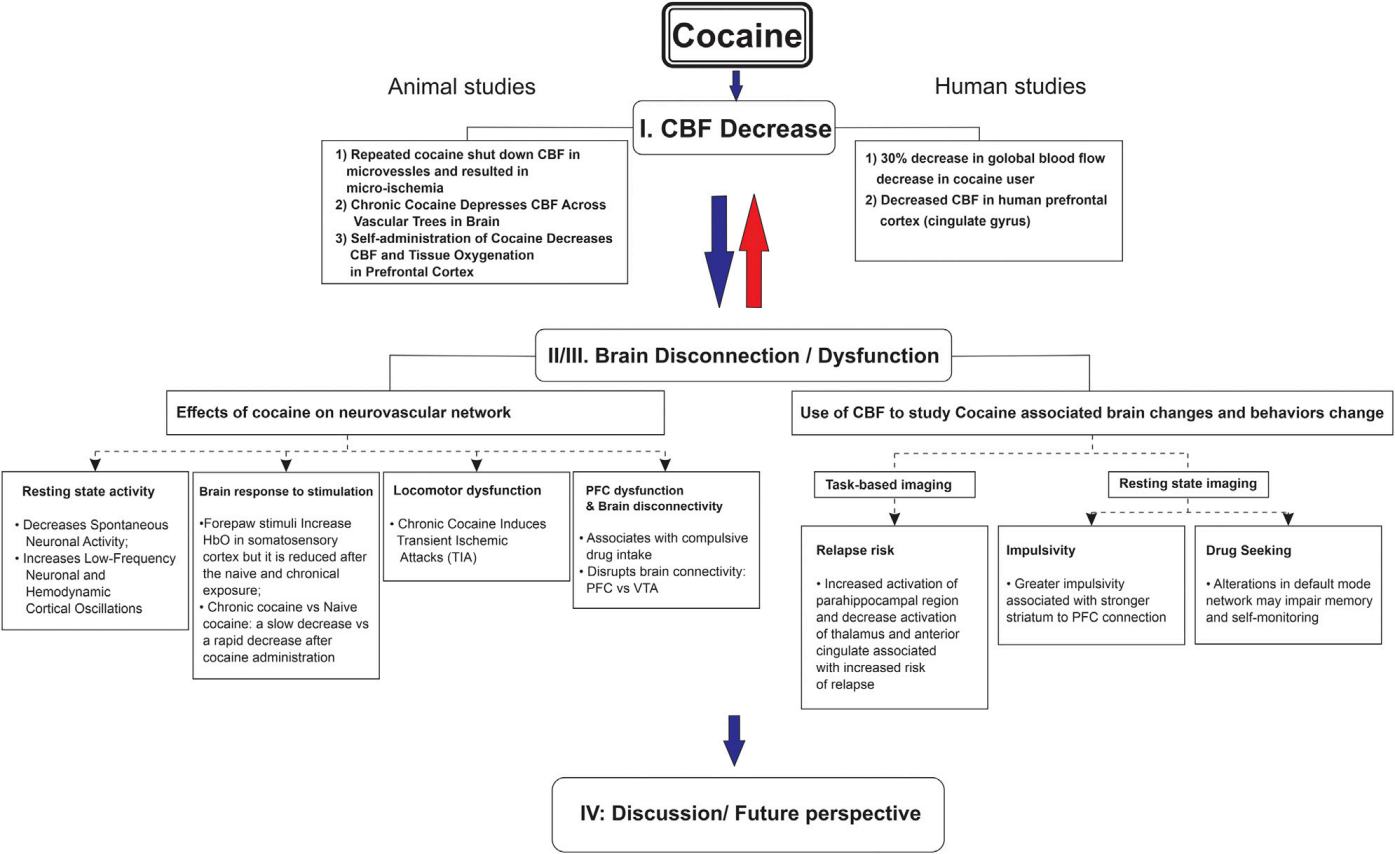
Cocaine-induced cerebral blood flow decrease associated with brain dysfunction from neurovascular imaging to human addictive behavior.

The goal of this literature review was to explore cocaine’s influence on CBF and whether it was associated with brain metabolic and/or functional changes, including long-term neurological complications. To conduct this review, we searched through publications in both preclinical and clinical research through the databases (PubMed, Embase, and Web of Science) focusing mostly on the past 10 years (2013–2023). The details of search keywords and the numbers of relevant articles identified are listed in Table 1, including our publications. Additionally, to ensure identification of all relevant literature, we utilized Chat GPT 3.5 to identify studies we might have missed. The major findings from these articles are summarized in Figure 1.

**TABLE 1:** Search strategy and outcome.

	PubMed		Web of science		Embase					
Aim	Approach	Identified	Approach	Identified	Approach	Identified	Relevant		Expert identified	Our involvement/publications
Cocaine- induced brain dysfunction with blood flow defects Or Effects of cocaine-induced blood flow reduction on brain function	((cocaine) AND (brain function)) AND (blood flow)	45	((ALL=(cocaine)) AND ALL=(brain function)) AND ALL=(blood flow)	34	(“cocaine”/exp OR “cocaine” OR “cocaine dependence”/exp OR “cocaine dependence”) AND (“brain function”/exp) AND (“brain blood flow”/exp)	25	Animal	18	1	12
							Human	7		
	(transient ischemic attack) AND (cocaine)	7	(ALL=(transient ischemic attack)) AND ALL=(cocaine)	5	(“cocaine”/exp OR “cocaine” OR “cocaine dependence”/exp OR “cocaine dependence”) AND (“Transient Ischemic Attack”/exp)	47	Animal	4	2	2
							Human	3		
	(cocaine) AND (neurovascular coupling)	4	(ALL=(cocaine)) AND ALL=(neurovascular coupling)	5	(“cocaine”/exp OR “cocaine” OR “cocaine dependence”/exp OR “cocaine dependence”) AND (“neurovascular coupling”/exp)	5	Animal	2	10	2
							Human	0		
	(cocaine) AND (self-administration) AND (blood flow)	6	((ALL=(cocaine)) AND ALL=(self administration)) AND ALL=(blood flow)	10	(“cocaine”/exp OR “cocaine” OR “cocaine dependence”/exp OR “cocaine dependence”) AND (“drug self administration”/exp) AND ('brain blood flow'/exp)	3	Animal	6	17	3
							Human	1		
	(cocaine) AND (brain response) AND (Resting state)	16	((ALL=(cocaine)) AND ALL=(brain response)) AND ALL=(resting state)	39	(“cocaine”/exp OR “cocaine” OR “cocaine dependence”/exp OR “cocaine dependence”) AND “resting state network”/exp OR “functional magnetic resonance imaging”/exp) AND (“brain blood flow”/exp)	17	Animal	1	18	1
							Human	9		
	(prefrontal dysfunction) AND (cocaine intake)	16	(ALL=(prefrontal dysfunction)) AND ALL=(cocaine intake)	12	(“prefrontal cortex”/exp) AND (“cocaine”/exp OR “cocaine dependence”/exp) AND (“hypoactivity”/exp)	8	Animal	6	11	1
							Human	3		
	((brain reactivity) AND (stimulation)) AND (cocaine)	29	((ALL=(brain reactivity)) AND ALL=(stimulation)) AND ALL=(cocaine)	23	(“evoked cortical response”/exp OR “self stimulation”/exp OR “deep brain stimulator”/exp OR “electrical brain stimulation test”/exp) AND (“evoked cortical response”/exp) AND (“cocaine”/exp OR “cocaine” OR “cocaine dependence”/exp OR “cocaine dependence”)	6	Animal	3	1	1
							Human	11		

## 3 Cocaine decreases cerebral blood flow

Cocaine’s toxicity and vascular effects have been investigated for more than 3 decades. A recent review (Bachi et al., 2017), proposed that acute cocaine-induced vasoconstriction is due to endothelin-1 release and a decrease of nitric oxide (a blood vessel dilator) (Saez et al., 2011; Saez et al., 2014). Cocaine-induced vasoconstriction caused deformation and damage of blood vessels, reducing cerebral blood flow (CBF). For example, cocaine exposure reduced cerebrovascular pulsatility and CBF in mice assessed with two-photon microscopy (Chen et al., 2020), and cocaine-induced CBF decrease was also detected in non-human primate subjects with positron emission tomography (PET) imaging (Howell et al., 2002). Additionally, a study using a two-sensor electrochemical recording method (Thomas et al., 2021) reported cocaine decreased oxygen levels in the nucleus accumbens (NAc) of rodents.

We have used advanced multimodality optical imaging techniques (including multi-wavelength optical imaging and optical coherence tomography) to study the effects of acute and chronic cocaine on vascular networks in the rodent brain using noncontingent and self-administration models. This multimodality optical approach allowed us to perform high-resolution *in-vivo* imaging of cerebral vessels; quantification of CBF and metabolism by assessing oxygenated and deoxygenated hemoglobin; and to record neuronal activity by measuring changes in intracellular calcium. Our *in-vivo* preclinical studies have led to the following findings.

### 3.1 Cocaine curtailed CBF velocity in microvessels and led to microischemia with repeated exposure

Ultrahigh-resolution Optical Doppler Tomography (μODT) (Ren et al., 2012a; Ren et al., 2012b; You et al., 2014) enabled tracker-free three-dimensional (3D) angiography of cerebral microvasculature. To quantify simultaneous changes in the vascular network and CBF in mice expose to cocaine, we implemented a phase-intensity-mapping algorithm (Yuan et al., 2011). When analyzed using the enhanced 3D μODT, microvessels in the mouse cerebral cortex demonstrated a 70% decrease in CBFv 2–3 min after acute cocaine administration. Following this reduction, the recovery varied by vessel type (venules: 12 min, arterioles: 5 min) with enhancement of these changes following chronic cocaine treatment. Interestingly, capillaries were unique in that their time to recovery ranged from 4–20 min. While no hemorrhage was observed in association to these vascular changes, repeated cocaine administration worsened local ischemia and vasoconstriction (Ren et al., 2012a). The response of cerebral vessels to cocaine supports its role in the development of cerebral micro-ischemia. Coupled with repeated injury due to chronic cocaine consumption, the ischemic environment may contribute to cocaine’s neurotoxic effects.

### 3.2 Chronic cocaine reduced CBFv across cerebral vascular trees

The cerebral microvascular networks (including capillary beds) along with larger-size branch vessels can be imaged with μODT, enabling quantification of CBFv changes (Ren et al., 2012a; Pan et al., 2014; You et al., 2014). Comparison of μODT images between control mice (not exposed to cocaine) and mice exposed to chronic cocaine showed dramatic flow decreases with chronic cocaine exposure (You et al., 2014). Specifically, CBFv decreased 22.5% ± 8.7% (*p* < 0.05) in arterial vessels, 68.4% ± 6.0% (*p* < 0.05) in venules and 49.1% ± 17.9% in the capillary networks. Similar experiments in rats showed that chronic cocaine exposure over 2–4 weeks significantly decreased CBFv across the cerebrovascular network in the somatosensory cortex (Zhang et al., 2016). A comparison between the two cocaine groups showed that CBFv in small vessels was significantly lower after 4 weeks than after 2 weeks of cocaine exposure. Therefore, these results show that chronic cocaine decreased CBFv across the vascular tree of various vessel sizes in the cerebral cortex of mice and rats.

### 3.3 Self-administration of cocaine decreased CBFv and tissue oxygenation in the prefrontal cortex

Rodent models of cocaine self-administration have been used to study addiction-like behaviors. In fact, methods that lead to high levels of cocaine intake in rats have been shown to be relevant to cocaine addiction in humans (Roberts et al., 2007). Behaviors associated with cocaine addiction, including tolerance, increase intake, and motivation are emulated in a rodent model that gives unrestricted extended access for cocaine self-administration. (Koob and Bloom, 1988; Koob and Volkow, 2010). Further emphasizing the transition from drug use to addiction, Vanderschuren and Everitt highlighted that drug-seeking behaviors in rodents become particularly compulsive after extended access to cocaine (Vanderschuren and Everitt, 2004).

Substance use disorder (SUD), as defined by DSM 5, includes compulsive behavior where the subject continues to invest significant amount of time seeking and consuming a substance despite awareness of the adverse consequences (Association, 2000; Association, 2013). Compulsive behavior is also being considered for inclusion into the International Diagnosis Codes (ICD-11) (Matone et al., 2022). Compulsive drug consumption is recapitulated in the Long-access (LgA; a daily 6-h of access session) self-administration model. Specifically, compared to a more time restricted access to the drug model (Short-Access or ShA, e.g., a daily 1-h session), extended access leads to increased cocaine intake and cocaine seeking (Wee and Koob, 2010). We conducted quantitative CBFv and angiographic imaging of neurovascular networks in the prefrontal cortex (PFC) of LgA animals and compared to yoked control (i.e., saline infusion instead of cocaine). We found that CBFv in the PFC of the LgA animals were much lower than in controls throughout the cerebrovascular tree. Moreover, CBFv in the PFC was negatively associated with cocaine doses self-administered by the animals, such that rats who consumed the largest doses had the greatest CBFv decreases (Du et al., 2020). These findings of CBFv reductions in PFC of animals undergoing self-administration of large cocaine doses are consistent with those observed in cocaine-addicted individuals.

The optical imaging findings revealed sensitization to cocaine-induced CBFv reductions, such that an acute cocaine challenge triggered significantly larger and longer-lasting CBFv reductions and Δ[HbO_2_] decreases in LgA than in drug naïve animals. Indeed, cocaine-induced decreases in Δ[HbO_2_] in LgA animals were double to those in controls (i.e., 20.7% *versus* 10.8%). Human studies with near infrared optical imaging have revealed that decreases in brain oxygen saturation of 13% were associated with EEG changes characteristic of cerebral ischemia in 97% of cases (Al-Rawi and Kirkpatrick, 2006). Considering that chronically exposed animals had a 20.7% reduction in Δ[HbO_2_] after acute cocaine dose, this indicates that these reductions are well within clinical values associated with ischemia.

Clinical studies have also reported that chronic cocaine exposures can cause neurological complications such as stroke. Chronic cocaine misuse has been consistently shown to lead to deficits in CBF including cortical perfusion (Volkow et al., 1988; Kosten, 1998; Lukas, 2014). Both positron emission tomography (PET) and single photon emission computed tomography (SPECT) studies have shown that chronic cocaine misusers had perfusion deficits that appeared similar to those seen after a stroke or head injury (Levin et al., 1994). The abnormalities in CBF have been associated with alterations in neuropsychological function and neurologic symptoms in cocaine misusers (Pascual-Leone et al., 1991a; 1991b; Strickland et al., 1993; Strickland et al., 1998; Bartzokis et al., 1999) including cocaine-induced strokes even among young misusers (Desai et al., 2017). The mechanism underlying cocaine-induced reductions in cerebral blood flow is not fully understood, yet studies have documented negative effects of cocaine on blood vessels, including endothelial damage and vasculature alterations, which are likely to contribute to neurotoxicity (Dressler et al., 1990; Diercks et al., 2008; Finkel and Marhefka, 2011).

## 4 Effects of cocaine on neurovascular networks

Cocaine-induced vasoconstriction reduces cerebral blood flow, but further reductions could occur through cocaine’s neuronal effects, which may affect neurovascular coupling (Ren et al., 2012a; You et al., 2017; Du et al., 2020). Optical imaging has made it possible to investigate in greater detail the effects of cocaine on neurovascular coupling (Ren et al., 2012a; You et al., 2017; Du et al., 2020).

### 4.1 Cocaine’s effects on resting-state brain activity

Multiple imaging modalities have been developed to investigate brain function using different methods to quantify neuronal firing and hemodynamics. Functional MRI (fMRI) utilizes the magnetic properties of hemoglobin to assess neuronal activity through the BOLD signal whereas tools such as electrophysiology directly measure local field potentials (FP) within a neuron. Additionally, optical technologies such as optical intrinsic signal imaging (OISI) and optical intracellular calcium fluorescence imaging (OFI) quantify neuronal and hemodynamic activity using light. The dynamic activity recorded by these different methodologies can be decomposed into unique oscillations at specific frequencies. All of these technologies demonstrate underlying Low-frequency oscillations (LFOs) in spontaneous neuronal activity and cerebral hemodynamic fluctuations (fMRI: (Fransson, 2005), OISI: (Du et al., 2014; Rayshubskiy et al., 2014), OFI: (Du et al., 2014; Vincze et al., 2018), FP: (Obrig et al., 2000; Buzsaki and Draguhn, 2004; Rayshubskiy et al., 2014)).

The generation of functional connectivity brain maps obtained during resting state conditions is based on the assumption that LFOs in neuronal activity are reflected in the hemodynamic LFOs as the neuronal and cerebral vessels are thought to be tightly linked through neurovascular coupling (Biswal et al., 1995; Fair et al., 2007; Raichle and Snyder, 2007). A study that used single electrode recording and optical intrinsic signal imaging showed that resting-state hemodynamic oscillations were regionally coupled to the multiunit activity (MUA) of a neuronal population (Ma et al., 2016). To assess if cocaine disrupted neurovascular coupling between neuronal and vascular LFOs, we simultaneously measured LFOs of neuronal activity and hemodynamics in naïve and chronic cocaine rats. Animals were tested at baseline and after acute cocaine. Specifically, we measured local field potentials and cerebral blood flow LFOs (0–1 Hz) in the somatosensory cortex of naïve and chronic cocaine (2 weeks) rats at baseline and during cocaine intoxication.

Our study showed that cocaine reduced spontaneous neuronal firing rates while synchronizing neuronal activity and enhancing LFOs in the somatosensory cortex. Reduced spontaneous neuronal firing with cocaine was consistent with prior findings from our group (Chen et al., 2016) and that of others (Bekavac and Waterhouse, 1995). The reduction in spontaneous neuronal activity has been interpreted to reflect cocaine’s dopaminergic enhancing effects (perhaps also its noradrenergic actions) (Bunney et al., 1973; Ritz et al., 1987; Einhorn et al., 1988). In the PFC, dopamine (DA) has been shown to suppress spontaneous neuronal firing of pyramidal neurons, an effect that was enhanced by cocaine (Kroener et al., 2009). By decreasing neuronal background firing, stimulants, such as cocaine, may improve detection of salient stimuli leading to enhanced attention. (Volkow et al., 2008; Arnsten, 2009). However, with chronic cocaine exposure, the suppression of spontaneous neuronal activity in the ventromedial PFC or anterior cingulate cortex could also contribute to compulsive cocaine intake in addiction by promoting inflexible behaviors (Volkow et al., 2011). An enhanced signal-to-noise ratio to a conditioned stimulus (cocaine or its cues) could produce an accentuated response to it while reducing the influence of weaker competitive stimuli. This would further strengthen conditioning while reducing the salience of nonrelated drug stimuli accounting in part for the behavioral inflexibility observed in individuals suffering from addiction (Wise and Kiyatkin, 2011; Volkow et al., 2013).

In addition to depressing spontaneous neuronal spiking activity, acute cocaine synchronized the fluctuation of the FP events in the LFO band (0.03–0.15 Hz), narrowing the power spectral distribution of the FP event rate. In naïve animals, cocaine depressed neuronal oscillation frequencies >0.15 Hz that recovered 20 min following cocaine administration. Chronically treated animals had a similar suppression of frequencies > 0.15 Hz following a cocaine challenge but the suppression was more prominent and persisted for up to 30 min. At baseline, chronically treated animals had accentuated LFOs suggesting that the effects of cocaine persisted up to 24 h after last injection. These findings are consistent with previous reports in the globus pallidus of increases in LFOs (0.017–0.1 Hz) in neuronal activity in the presence of cocaine and other dopamine agonists (Ruskin et al., 1999).

We observed that while in naïve animals the effects of acute cocaine on CBF fluctuation spectra were similar to those on neuronal activity, in the chronic animals, acute cocaine further narrowed the spectral distribution of neuronal LFOs but it did not further change hemodynamic LFOs. We interpreted this to reflect decreased hemodynamic reactivity to further changes in neuronal activity in chronic cocaine animals. This neurovascular uncoupling might reflect the marked and persistent vasoconstriction associated with chronic cocaine exposure (Yin et al., 2017), which might limit the hemodynamic responses associated with neuronal oscillations.

In summary, acute and chronic cocaine administration depressed neuronal activity in the somatosensory cortex but increased LFP oscillations in neuronal activity and CBF fluctuations. The changes of neuronal LFOs were spectral-temporally correlated with the changes of CBF LFOs in the resting state in naïve animals, demonstrating neurovascular coupling in response to an acute cocaine challenge. In contrast, the LFO changes in neuronal activity and CBF fluctuations deviated from a linear relationship in chronic cocaine-exposed animals, suggestive of hemodynamic uncoupling from neuronal activity under resting state conditions.

### 4.2 Cocaine’s effects on brain response to stimulation

Many researchers have studied cocaine effects at the molecular level including in proteins (Martinez et al., 2014; Jin et al., 2015; Wiskerke et al., 2016; Hurley et al., 2017; Coyle and Balu, 2018), genetics/epigenetics (Hachem-Delaunay et al., 2015), and their relationship to behavior (Antoniazzi et al., 2014; Lespine et al., 2019). However, cocaine’s effects on neurovascular networks and their functional consequences have not been fully investigated.

Functional MRI allows for the study of regional patterns of brain activation triggered by tasks, stimuli, and drugs such as cocaine. Activation and deactivation as assess by BOLD in fMRI assumes that there is a strong correlation between neuronal and hemodynamic activity through neurovascular coupling (Logothetis et al., 2001; Li and Freeman, 2007). Cocaine, a highly vasoactive substance, interferes with the tight coupling between neurons and cerebral vessels and may confound data collected on neuronal activation through BOLD (Iannetti and Wise, 2007). One study by Gollub et al. found that while, the fMRI measurements of the visual cortex before and after cocaine administration showed no change in BOLD, the use of the FAIR (flow-sensitive alternating inversion recovery) method demonstrated decreased CBF (Gollub et al., 1998). Consistent with the reduction in CBF seen with FAIR, studies have also found decreased functional connectivity, which was interpreted as a reduction in neuronal activity (Li et al., 2000). Therefore, cocaine effects on cerebrovascular reactivity may complicate the interpretation of BOLD contrast fMRI as well as results from other imaging studies that rely on CBF measurements.

To distinguish cocaine-induced vascular effects from its neuronal actions, we combined Laser Doppler Flowmetry (LDF) with electroencephalography (EEG) to simultaneously measure CBF (reflecting neurovascular hemodynamics) and local field potential (LFP, reflecting neuronal activities) in the rat somatosensory cortex (Chen et al., 2016) during resting state (i.e., non-stimulation state) and during electrical forepaw stimulation before and after acute cocaine. Neuronal activity was random during the resting state but synchronized during forepaw stimulation. Cocaine depressed both resting-state LFP and CBF and these changes were strongly correlated with each other (r = 0.81, *p* < 0.001) supporting that neurovascular coupling was maintained. In contrast, acute cocaine disrupted the neurovascular coupling following forepaw stimulation, as the CBF increase was attenuated by 50% for ∼20 min. The disruption of neurovascular coupling following forepaw stimulation but not in the resting state, suggests that distinct mechanisms may control neurovascular regulation in response to stimulation as compared to at rest. Alternatively, it might reflect that the magnitude of the spontaneous flow increase while at rest is much smaller than during activation. Therefore, despite the vasoconstriction, the vessels can still accommodate the small spontaneous flow change but not the larger increases with stimulation. Our studies demonstrate that when interpreting fMRI studies in chronic cocaine users and controls, it may be necessary to consider the hemodynamic and vascular state as CBF changes in response to stimuli may be dependent on background CBF conditions.

To further characterize chronic cocaine’s effects, we measured the CBF responses along with oxygenated-hemoglobin (HbO_2_) and deoxygenated-hemoglobin (HbR) dynamics to forepaw electrical stimulation in naive and chronic cocaine treated animals. Our experiments showed that stimulation-evoked tissue HbO_2_ response decreased after cocaine in both control (naïve) and chronic groups compared to their baselines (prior to acute cocaine administration) and did not recover until 28 min post cocaine. The HbO_2_ decrease in the chronic cocaine group was slower (minimum value ∼16 min after cocaine) than in the control group (minimum value at 4–8 min). A CBF decrease was also observed after acute cocaine that partially recovered in the control group at 28 min and fully recovered in the chronic group at around 20 min. However, the chronically treated group had lower basal CBF (presumably due to persistent vasoconstriction), thus, even after recovery from acute cocaine the CBF was still lower than the basal levels in control rats. Similarly, hemoglobin responses were weaker in the chronic cocaine group compared to the control group during the baseline period, which indicated that chronic cocaine accentuated neurovascular decoupling and dysfunction. (Du et al., 2020).

Taken together, these findings revealed that cocaine attenuated blood flow and depressed spontaneous neuronal activity at rest. Following stimulation, it did not change the neuronal response but reduced CBF responses, indicating that neurovascular coupling during stimulation was temporarily disrupted by cocaine. Neurovascular uncoupling could contribute to cocaine’s neurotoxicity, particularly for stimulation conditions when CBF might be insufficient to sustain the energetic demands of neuronal tissue (Chen et al., 2016).

### 4.3 Chronic cocaine interferes with cerebrovascular physiology

The risk of cocaine use leading to cerebrovascular complications has been extensively reported (Treadwell and Robinson, 2007; Cheng et al., 2016). Cocaine intake activates the sympathetic system, leading to hypertension, tachycardia, and increased metabolic demand, all of which can contribute to vascular events (Daras et al., 1994). Such activation is particularly concerning given that hypertension is a known risk factor for cerebrovascular diseases.

Cocaine can also disrupt the integrity of the blood-brain barrier, making the central nervous system more susceptible to toxins and potentially leading to inflammatory responses (Fiala et al., 1998). The blood-brain barrier acts as a protective shield, preventing harmful substances from entering the brain. Disruption of this barrier could lead to increased susceptibility to infections and other pathologies.

Chronic use of cocaine has been associated with arterial stiffness, which would further enhance the risk of cerebrovascular accidents. Arterial stiffness makes blood vessels less capable of expanding and contracting, decreasing flow (Kozor et al., 2014). This in turn increases the risk of ischemic events as reduced flow can starve brain tissues of essential oxygen and nutrients.

In the context of treatment, understanding these cocaine-induced cerebrovascular changes is crucial. Use of neuroimaging, including fMRI, has allowed investigators to monitor real-time brain activity and vascular responses relevant to cocaine’s cerebrovascular effects (Kaufman et al., 1998).

In conclusion, it is clear that cocaine use poses a significant risk to cerebrovascular health, not just because of its direct effects but also due to the cascade of physiological responses it triggers. Comprehensive studies, innovative imaging techniques, and a deeper understanding of the physiological changes induced by cocaine are paramount in designing effective treatments and interventions.

### 4.4 Cocaine disrupted brain connectivity

The PFC is modulated by dopaminergic and glutamatergic neurons that project from the ventral tegmental area (VTA) and its disruption might facilitate impulsive behaviors as occurs during cocaine intoxication. The involvement of the dorsal lateral PFC and anterior cingulate cortex (ACC) in self-regulation and decision making and its relevance to impulsive behaviors (Goldstein and Volkow, 2011) and addiction has been extensively investigated (Baler and Volkow, 2006). To study this, we assessed the effects of acute cocaine (30 mg/kg, i.p.) on the reactivity of the PFC to VTA stimulation (Park et al., 2022) using a genetically encoded calcium indicator (GCaMP6f), which allowed us to track neural activity (Chen et al., 2013), in medial PFC (mPFC) in response to “tonic-like” (5 Hz) and “phasic-like” (50 Hz) electrical VTA stimulation. The 5 Hz and 50 Hz stimuli were used to mimic DA neuronal tonic and phasic firing in the VTA, respectively (Schultz, 2007). We wanted to distinguish the effects of tonic dopamine firing (1–5 Hz), which contributes to low stable levels of extracellular DA necessary for sustained processing and motivation to those from phasic firing (40–50 Hz), which occurs in bursts and induces DA concentration spikes that are relevant to reward and conditioning (Grace and Bunney, 1984; Robinson et al., 2004; Schultz, 2016) including to drugs (Berridge and Robinson, 1998). In contrast, tonic stimulation of VTA DA neurons attenuates drug consumption (Bass et al., 2013). The high temporal and spatial resolutions of optical imaging techniques have greatly advanced our understanding of neural circuits (Knopfel, 2012). This includes giving researchers the ability to capture single neuronal Ca^2+^ transients from individual stimuli with “tonic-like” stimulation and neuronal activation evoked by “phasic-like” VTA stimulation.

Our results showed that “tonic-like” VTA stimulation induced a rapid increase in neuronal Ca^2+^ in mPFC followed by a plateau and recovery upon termination of stimulation. However, after cocaine, the mPFC sensitivity to “tonic-like” VTA stimulation was attenuated. Such perturbations in neural responsiveness might underlie the alterations in decision-making and heightened impulsivity during cocaine intoxication (Jentsch and Taylor, 1999). Impulsivity, is the tendency to act on impulse with no regard to consequence and is described as a lack of behavioral inhibition by DSM 5 (Association, 2013). As a multidimensional construct, impulsivity includes a range of “actions that are poorly conceived, prematurely expressed, unduly risky, or inappropriate to the situation and that often result in undesirable outcomes” (Daruna and Barnes, 1993). One particularly relevant dimension of impulsivity for understanding the neural impact of substance use involves the concept of disrupted inhibitory control and is strongly associated with substance use disorders (Jentsch and Pennington, 2014; Kozak et al., 2019). “Phasic-like” stimulation evoked a rapid Ca^2+^ fluorescence increases in the mPFC with an immediate decay process and cocaine further shortened the recovery time. Cocaine’s modulation of this response hints at its profound impact on reward-related neuronal processes (Hyman et al., 2006). These changes in mPFC might contribute to cocaine binging during intoxication and is consistent with findings of decreased tonic DA function in cocaine misusers (Volkow et al., 1997; Martinez et al., 2007; Volkow et al., 2014). This impaired PFC function could also contribute to drug-seeking and relapse (Everitt and Robbins, 2005).

### 4.5 Cocaine-induced ischemia in PFC is associated with escalation of cocaine intake

Chronic cocaine exposure in rats has been associated with loss of neurons in the PFC (George et al., 2008). Within the PFC, the prelimbic cortex (PrL) is involved in controlling drug-seeking behavior (Martin-Garcia et al., 2014), as is the orbitofrontal cortex (OFC) (Limpens et al., 2015). Reduced neural activity in PrL and OFC have been linked with persistent seeking behaviors and are likely to contribute to compulsive aspects of addiction (Limpens et al., 2015).

Deficits in PFC including OFC and dorsal ACC (analogue of rodent PrL) are associated with the development of compulsive cocaine use in humans (Rapinesi et al., 2016). Chronic cocaine consumption alters the normal striato-cortical circuitry leading to prefrontal dysfunction (Volkow and Morales, 2015). In additional to the neural circuitry adaptions, cocaine-induced alterations of CBF and the cerebrovasculature are also likely to play a role in hypofrontality.

Indeed, cocaine misusers are at higher risk of ischemic and hemorrhagic strokes in the brain than non-users (Levine et al., 1987; Tuchman et al., 1987; Levine et al., 1991) and imaging studies in cocaine misusers have documented marked decreases in CBF, which are most prominent in PFC (Volkow et al., 1988).

As discussed in prior sections, rodent studies have shown that chronic cocaine triggers vasoconstriction, reduces CBF, and results in cerebral ischemia (Zhang et al., 2016). By applying integrated optical imaging to investigate these phenomena, we documented that despite significant proliferation of blood vessels in areas of vasoconstriction (Zhang et al., 2016; Allen et al., 2019; Du et al., 2020), CBF remained reduced even after 1 month of cocaine detoxification (Du et al., 2020). This could explain why deficits in executive control can persist in cocaine users even months after drug discontinuation (Rezapour et al., 2016).

## 5 Clinical studies of cocaine’s impact on CBF

It has been well established that chronic use of cocaine leads to global and regional CBF decreases in the brain. Volkow et al. using PET first reported on this finding, revealing that chronic cocaine users had significant decreases in CBF relative to non-users (Volkow et al., 1988). Following this work, Wallace et al. quantified the change in relative CBF following 40 mg of intravenous (IV) cocaine in patients with cocaine use disorder (CUD). These patients demonstrated CBF reductions of 30% within the anterior cingulate cortex (Wallace et al., 1996). As summarized in Table 2, recent literature has replicated these findings. For example, Luo et al. used a stop signal task to study the impact of cocaine on brain regions involved in error processing and how these changes may predict relapse. They found that decreased flow to higher level processing centers of the brain such as the dorsal ACC, predicted early relapse following discharge (Luo et al., 2013). This is consistent with the findings reported above in preclinical models of attenuated blood flow in PFC of chronically treated animals.

**TABLE 2:** Clinical evidence of cocaine’s impact on CBF.

Author (year)	Study type	Subjects and reported average usage	Objective	Outcome
Volkow et al. (1988)	Prospective Cohort	27 cocaine abusers; average age 27	To determine cerebral blood flow dynamics in cocaine abusers using PET	Cocaine users demonstrated alter isotope uptake in frontal cortex indicative of impaired blood flow.
Wallace et al. (1996)	Prospective Cohort	4 cocaine abusers; age range from 31–34; Average cocaine use period of 10 years; Average 3g/day	Assess relative change in cerebral blood flow in different brain regions of cocaine abusers using SPECT	Mean decease in CBF across whole brain was 30% Quantified relative decrease in CBF in different brain regions.
Luo et al. (2013)	Prospective Cohort	97 cocaine abusers; age range from 18–55; 17.1 average years of cocaine use; 9.9 g of cocaine in last month consumed	Determine if male and females have gender specific predictors of relapse	Decreased activation of the thalamus and dorsal anterior cingulate cortex was associated with relapse in females. Males who relapsed sooner had lower activation of the dorsal anterior cingulate and left insula.
Forster et al. (2018)	Meta-analysis	24 studies with 923 subjects	Identify markers of probable relapse and abstinence utilizing PET and fMRI imaging	Activation of right putamen with drug cue presentation was associated with an increased risk of relapse. Activation of right anterior cingulate cortex to cues was associated with increase abstinence.
Adinoff et al. (2015)	Prospective Cohort	40 cocaine abusers; 20 matched controls; average age 42.2	Utilized BOLD resting state functional connectivity and CBF to predict relapse following treatment for CUD	Increase probability of relapse in patients with increased cerebral blood flow in left posterior hippocampus (pHp). Increase connection strength between pHp and posterior cingulate cortex was also associated with relapse.
Hu et al. (2015)	Case-control	56 cocaine abusers and 56 healthy controls; average age cocaine abusers 39.8 years; average cocaine use of 12.6 years	Determine if cocaine abusers have altered functional connectivity in the striatal circuitry	Increased functional connectivity between the striatum and the dorsal lateral PFC was associated with amount of cocaine use and impulsivity.
Berlingeri et al. (2017)	Case-control	18 cocaine abusers abstinent for 5 months; average ag 39: average time of abstinence 141 days; 19 control patients	Determine if resting state fMRI can determine cocaine-induced dysfunction in reward regions following a period of abstinence	Abstinent cocaine abusers had increased functional connectivity between NAc and dorsal striatum as well as a reduction between NAc and dorsal PFC.
Ray et al. (2016)	Case-control	20 cocaine abusers undergoing 72 h abstinence; Average age 46; Average length of cocaine use 16 years; 17 control patients	Investigate functional connectivity in the mesocorticolimbic DA system in chronic cocaine users	Functional connectivity between the VTA and NAc, hippocampus, and mPFC was increased in recently abstinence cocaine users as compared to healthy control.

### 5.1 Relapse and remission

A recent meta-analysis looking at functional studies from 2000–2017 using CBF to identify brain structures involved in relapse and remission identified only 3 papers that specifically focused on cocaine use disorders (CUD) (Forster et al., 2018). These papers identified different regions whose activation or inhibition predispose patients to increased risk of relapse or remission. Activation of the left posterior hippocampus as determined by relative CBF increases was the only difference observed in voxel-wise whole brain analysis in CUD patients that predicted relapse within 30 days of discharge (Adinoff et al., 2015).

In contrast, Luo et al. reported sex-specific decreases associated with relapse. In females, attenuated dorsal ACC and thalamus activity predicted earlier relapse, while in males, it was the decreased activity in left insula and ACC (Luo et al., 2013). The likelihood of relapse was predicted with an area under the curve of 0.85 for a receiver operating analysis. Another review by Jasinska et al. undertook a comprehensive survey of human neuroimaging results, pointing out factors modulating neural reactivity to drug cues in addiction, including cocaine (Jasinska et al., 2014).

### 5.2 Alterations in resting state of cocaine users and PFC dysfunction

While rodent studies have help determine how different subregions, of the PFC contribute to the development of CUD, translating these findings from rodents to humans has been challenging as the rodent networks need to be identified and mapped to the human brain (Box 1). Therefore, to determine how chronic cocaine use, abstinence, and relapse alters normal human brain function, clinical functional imaging studies have investigated resting functional connectivity. Hu et al., used fMRI to measure resting functional connectivity in 56 patients with CUD and reported that patients with higher impulsivity scores (BIS-11) or who used cocaine recently had increased connectivity between the striatum and dorsal lateral PFC (Hu et al., 2015).

There is increased recognition that bi-directional signaling between the PFC, nucleus accumbens (NAc), and VTA are crucial to drug reward and addiction. Significant alterations in this network have been reported in CUD. For example, whereas former patients with CUD showed decreased connectivity between the right NAc and the dorsal PFC (Berlingeri et al., 2017), increased connectivity between PFC and VTA and between NAc and mPFC was found in non-treatment seeking individuals with CUD (Ray et al., 2016). Insights by Volkow et al. further supported the notion of unbalanced neuronal circuits in addiction, with enhanced reactivity of circuits associated with saliency, emotion, and reduced function of executive networks in cocaine misusers (Volkow et al., 2013).

BOX 1 **Prefrontal cortical correlates between rodents and humans**

In order to study the neurobiological changes associated with the transition from voluntary to compulsive use, rodent models have been heavily utilized as a model of addiction. These pre-clinical studies have provided insights into the functional role of specific brain regions and their contribution to CUD and have implicated the PFC in the neurobiology of addiction including a role in risk for drug use and substance use disorders.

Different strategies have been employed to try to determine homology between rodent and human PFC based on functional connectivity or on cellular structure (Heilbronner et al., 2016; Preuss, 1995). Within the rodent PFC, there are four main structures of interest for drug addiction: the anterior cingulate gyrus, the prelimbic cortex, the infralimbic cortex, and the orbitofrontal cortex.

The anterior cingulate gyrus in rodents is thought to correspond to Brodmann areas 24, 25, and 32 in humans and to contribute to attention allocation, and salience (Paus, 2001; Ceceli et al., 2022). In BOLD imaging, ACC activation was found to be positively associated with strength of cocaine craving with exposure to cocaine-associated cues (Goldstein et al., 2009).

The medial prefrontal cortex in the rodent brain can be further subdivided into the Prelimbic (Prl) and infralimbic (IL) cortex. Studies suggest that these two regions play antagonistic roles with the Prl contributing to cocaine seeking and relapse whereas, the IL works to inhibit actions and promote drug extinction (Nett and LaLumiere, 2021; Moorman and Aston-Jones, 2023). Moreover, while the homolog of the IL in humans has been identified as the ventromedial prefrontal cortex (Brodmann area 25), there appears to be no clear correlation for the Prl (Nett and LaLumiere, 2021). Depending on the study, the Prl is either correlated with the dorsal lateral prefrontal cortex (Brodmann are 9 and 46) or the AAC in Brodmann area 32 (Heilbronner et al., 2016).

Lastly, the orbitofrontal cortex, whose homolog is the orbital frontal cortex (Brodmann areas 10, 11, 47) is involved with prediction and prediction error (Stalnaker et al., 2015). This brain region is thought to integrate different possible outcomes given a situation an individual has never experienced before (Levy and Glimcher, 2012). Following the event, the OFC might process the outcome and determine where errors were made in its prediction calculation in a process named “Credit error” (Lak et al., 2014). In addition, impaired OFC processing may lead to incorrect value attribution and the decision to pursue cocaine triggering relapse.

### 5.3 Limitations of clinical Studies

While current clinical studies have provided important insight into CUD pathology and the underlying changes in brain functional activity and CBF, there are limitations shared by these investigations. One limitation is the relatively small samples size of these clinical neuroimaging studies such that the largest size of the reviewed studies included 97 patients (Table 2). Thus, pooling of data through meta-analysis, is necessary to draw more robust conclusions. These studies are also confounded by variability in length of cocaine use prior to the investigation. Patients within the same study may therefore have different degrees of functional changes associated with length of drug misuse. Lastly, clinical studies are limited in that they can only study misusers after a varying period of use. While pre-clinical studies can investigate the pathophysiology and neuropathology of addiction as it evolves from first drug exposure to compulsive use, clinical studies are unable to examine the early stages of addiction due to difficulties in identifying patients early in the transition from voluntary to compulsive use. Ongoing large prospective brain imaging studies of adolescents as they transition into adulthood will allow us to investigate brain patterns that might help predict future drug use and addiction (Volkow et al., 2018).

## 6 Involvement of astrocytes in neurovascular regulation and Cocaine’s actions

Changes in brain function and CBF associated with cocaine may reflect adaptations in neurovascular coupling, a dynamic and complex system which allows for communication between neurons and blood vessels and regulates what crosses into the brain via the blood brain barrier. The exact mechanism of communication between neurons and cerebral vasculature remains unclear. Some literature suggests that the demand for increased blood flow is driven by a deficit of energy resources such as ATP and oxygen while others suggest that it is triggered by intracellular signaling resulting from synaptic activity (e.g., Ca^2+^) (Attwell et al., 2010; Mishra, 2017).

Astrocytes are a critical component of neurovascular coupling uniquely positioned between neurons and the vasculature. Their end-feet wrap around cerebral vessels contributing to the blood brain barrier while their processes interact with neurons and other astrocytes to create a functional syncytium (Mishra et al., 2016). Previous investigations into how astrocytes influence vessel diameter have established that increases in astrocytic Ca^2+^ in response to neurotransmitters such as glutamate leads to the release of vasoactive compounds that influence cerebral vessel tone (Simard et al., 2003; Gordon et al., 2008; Mishra et al., 2016). One of these compounds, 20-hydroxytetraenoic acid (20-HETE), is hypothesized to stimulate vasodilation by allowing Ca^2+^ entry into vascular smooth muscle cells via L-type Ca^2+^ channels (Rousseau et al., 2005). These same channels have been previously shown to attenuate the reduction of oxygenated hemoglobin in rats exposed to chronic cocaine (Du et al., 2021). However, the relationship between vascular dynamics and astrocytic activity with cocaine exposure have not been clarified. We have performed a series of studies to test the role of astrocytic Ca^2+^ accumulation in cocaine effects in the vasculature including neurovascular coupling dynamics and shown that cocaine effects on astrocytic activity modulate vascular reactivity (Liu et al., 2022).

Taken together, the current literature on CUD supports tripartite pathophysiology with neuronal, vascular and astrocytic contributions. Because of astrocytes proximity and interaction with neurons and cerebral vessels, they present a possible target for therapeutic intervention. However, there is still a substantial gap in the literature regarding the role of astrocytes in regulative vasculature in CUD. At the time of writing, a PubMed search using the MeSH terms “Cocaine-Related Disorders,” “Astrocytes,” and “Cerebrovascular Circulation” yielded only one publication by Liu et al. In this investigation, the team utilized genetically encoded calcium indicators and chemogenetics to manipulate astrocytes and observed its impact on cocaine-induced changes. They found that while neurons had a greater increase in intracellular calcium following cocaine, astrocytic calcium stayed elevated longer and was correlated with the flow of oxygenated hemoglobin in cerebral arteries. Importantly, inhibition of astrocytes using DREADD (Gi) minimized cocaine-induced vasoconstriction (Liu et al., 2022). This data demonstrated that astrocyte activation is critical for modulating vascular reactivity to cocaine in the brain parenchyma.

Astrocytes are also uniquely integrated into the dopamine reward circuitry with subgroups of astrocytes only responding to dopamine-1 receptor (D1R) medium spiny neurons (MSNs) or to dopamine-2 receptor (D2R) MSNs (Martin et al., 2015; Graziane et al., 2016). Astrocytes are also directly sensitive to changes in dopamine as optogenetic stimulation of the VTA triggering the release of DA within the reward circuity increased astrocytic calcium via astrocyte D1R or inhibition of astrocytic calcium via astrocyte D2Rs (Xin et al., 2019; Corkrum et al., 2020). Changes in the structure of astrocytes have also been associated with specific drug behaviors. In rodent models of cocaine self-administration, astrocytes in the NAc had smaller volumes and surface area following extinction of self-administration; whereas astrocytes in animals that did not undergo extinction did not show changes (Testen et al., 2018).

## 7 Discussion and future perspectives

Both pre-clinical and clinical investigations of chronic cocaine have documented decreased CBF in PFC, which may contribute to impulsive and compulsive behaviors (Tables 1, 2). Studies provide evidence that both reduced DA signaling and adaptations in the cerebral vasculature contributes to hypofrontality (Volkow and Morales, 2015; Zhang et al., 2016). Indeed, in our preclinical studies increases in neuronal calcium and decreases in oxygenated hemoglobin during acute cocaine intoxication in PFC correlated with cocaine intake. Moreover, pretreatment with an L-type calcium channel blocker attenuated these effects and was associated with reduced cocaine self-administration. These results provide evidence that prefrontal dysfunction mediated by cocaine induced changes in vascular physiology and in neuron/astrocytic activity contribute to addictive behaviors, such as compulsive drug intake (Du et al., 2020).

### 7.1 Recovery of neurovascular coupling following abstinence in humans

While the deficits in neurovascular coupling and its contribution to addiction associated behaviors have been examined, there is limited clinical literature on the recovery of this system following abstinence. A systemic review in 2022 that focused on structural and functional recovery following abstinence identified only 5 longitudinal imaging studies on cocaine abstinence (Alper et al., 1998; Moeller et al., 2012; Balodis et al., 2016; Parvaz et al., 2017a; Parvaz et al., 2017b; Parvaz et al., 2022). These studies investigated functional changes following cocaine withdrawal using BOLD or EEG as well as grey matter volume.

An investigation of the effects of chronic cocaine in the main cerebral arteries (Middle Cerebral Artery (MCA), Anterior Cerebral Artery (ACA), and Posterior Cerebral Artery (PCA)) using transcranial doppler (TCD) reported that cocaine users had decreased flow velocity with increased pulsatility index (PI). This pattern is suggestive of cerebrovascular disease caused by increased resistance in the small cortical vessels consistent with pre-clinical observations. As described above, animals models of chronic cocaine show constriction, and ultimately loss, of small vasculature in the brain parenchyma. Interestingly, following 30 days of monitored withdrawal in a closed psychiatric facility, repeat TCD showed only minor improvement in MCA velocity and no change in PI indicative of lack of recovery in the cerebrovasculature following this short abstinence window (Herning et al., 1999). This is consistent with reported investigations in mice, which found no change in cerebral hemodynamics or neovascular density following 30 days of withdrawal after 1 month of cocaine treatment.

However, longer periods of abstinence have shown more promising results regarding functional recovery. In Connolly et al. short-term abstainers (2.4 weeks) and long-term abstainers (69 weeks) in a semi-closed recovery unit underwent functional MRI to investigate cognitive impact of long-term abstinence. Using a GO/NOGO task, patients cognitive performance was assessed including their ability to successfully inhibit and errors of commission. While both groups showed increased inhibitory activity, short-term abstinent patients had increased activity in the dorsal regions of the frontal gyri while the longer-term abstainers showed increased activity in the inferior gyri, an area strongly associated with response inhibition (Connolly et al., 2012). Although limited, current investigations suggest that there is improvement in prefrontal function following abstinence with decreasing levels of impulsivity. However, it remains unclear whether this is a recovery in neuronal or astrocytic function or more closely associated with a recovery in cerebral vasculature.

### 7.2 Effects of cocaine on the blood brain barrier

The Blood-Brain Barrier (BBB), composed of astrocytes and endothelial cells regulates the passage of substances between the circulation and CNS (Obermeier et al., 2013; Banks, 2016). This barrier protects the brain from pathogens and regulates homeostasis. Substances of abuse, such as cocaine, have been found to increase the permeability of the BBB, allowing for the entry of toxins and contributing to neurological diseases such as HIV-associated Neurocognitive Disorder (HAND) (Fiala et al., 2005; Kousik et al., 2012).

Current literature supports that cocaine’s effects on the BBB involve various pathways. For example, chronic cocaine alters the surrounding extracellular matrix and damages the basement membrane of endothelial cells through iNOS-dependent upregulation of metalloproteinases (Sharma et al., 2009; Smith et al., 2014; Ysrayl et al., 2019). Other studies have suggested that the degeneration of the BBB is associated with cocaine-induced activation of the platelet-derived endothelial growth factor pathway. Activation of this cascade triggers a proteolytic signaling ultimately increasing cellular membrane permeability (Yao et al., 2011; Pimentel et al., 2020). Lastly, glial cells have also been implicated in damaging the BBB through cocaine-induced increase in pro-inflammatory cytokines, which increase astroglial activation, thus decreasing BBB integrity (Yao et al., 2011; Yang et al., 2016). These same cytokines, which contribute to BBB damage, are also responsible for accelerating HAND, as these molecules increase recruitment of monocytic cells that carry the HIV virus into the brain parenchyma (Dhillon et al., 2008).

### 7.3 Neuronal-astrocytic interactions and their role in cocaine-induced toxicity and addiction

Though for a long time it was thought that astrocytes only provided structure and nutrients to surrounding neurons, there is a much better understanding of the diverse roles of astrocytes in brain function including regulation of the synaptic microenvironment and modulation of CBF in response to neural demand (neurovascular coupling). Astrocytes display unique signaling dynamics in which calcium oscillations can propagate between astrocytes that are thought to represent gliotransmission (Xu et al., 2010; Srinivasan et al., 2015; Hanani and Verkhratsky, 2021). The regulation of glutamate, a bidirectional neuronal and glial transmitter, has been specifically implicated in addictive behaviors. The development of drug seeking and reinstatement have been linked to disruptions in astrocytic glutamate within the NAc. Both chemogenetic and pharmacological manipulation of glutamate transporters ameliorated these behaviors (Kenny et al., 2005; Miguens et al., 2008; Li et al., 2018). Specifically, activation of excitatory DREADD (Gq) selectively expressed in astrocytes in NAC prevented cue-induced reinstatement following extinction (Scofield et al., 2015). It is hypothesized that astrocytes enable increased intrasynaptic glutamate to feed back onto presynaptic inhibitory glutamate receptors preventing overexcitation by limiting the release of this excitatory transmitter (Knackstedt et al., 2009). In the absence of astrocytic stimulation, such as with chronic cocaine exposure, the excess of synaptic glutamate from the loss of presynaptic mediated inhibition of release may contribute to cocaine’s neurotoxicity through glutamate mediated excitotoxicity (Olney et al., 1991).

Acute cocaine triggers global cerebral vasoconstriction and decreased CBF and chronic use leads to vascular remodeling including angiogenesis to compensate for hypoxic injury. Astroglia appear to regulate vascular tone as their chemogenetic inhibition vasodilates cerebral blood vessels and increases CBF. Following a cocaine challenge, their inhibition abolishes cocaine-induced vasoconstriction and attenuates the reductions in CBF while also limiting neuronal calcium influx (Pan et al., 2023). Given astrocytes control of the local synaptic environment and vascular tone, they offer a promising new target for treatment of cocaine-induced neuronal toxicity and hypoxia.

### 7.4 Translation of discoveries into treatments

Over the past 10 years, there have been significant investments into the development of treatments for CUD (Supplementary Table S1). Advancements in the treatment of CUD have focused on either stimulating or inhibiting various types of dopamine (DA) receptors (i.e., D1R, D2R, and D3R) through agonists or antagonist but findings are inconclusive. Following a similar strategy to that of methadone as a replacement for opioid use, the utilization of amphetamine to treat CUD patients has been explored. The increase monoamines triggered by amphetamine might decrease cocaine seeking by increasing tonic DA levels and preventing burst firing (Grace, 1991; Grabowski et al., 2004; Brandt et al., 2021). Indeed, clinical trials have shown that dextro-amphetamine decreased cocaine intake in CUD patients (Shearer et al., 2003). Other medications such as modafinil, which enhances DA by inhibiting DA transporter (Volkow et al., 2009), have demonstrated mixed results in a meta-analysis of 11 randomized control trials with seven studies showing no benefit over placebo (Sangroula et al., 2017).

Transcranial magnetic stimulation (TMS) of the prefrontal cortex has also been studied for its potential to treat CUD. TMS can impact cortical functional activity and long-term potentiation or depression (Sanna et al., 2019). Targeted high frequency stimulation of the prefrontal cortex of rodents’ triggers release of DA in striatal regions with this effect paralleled in clinical studies showing that prefrontal stimulation released DA in the ipsilateral caudate nucleus. Behaviorally, repeated high frequency stimulation of the dorsal lateral prefrontal cortex of patients with CUD has led to decreased rates of relapse and reduced cocaine craving (Terraneo et al., 2016).

In addition to therapeutics that directly target the DA system, other pharmacological non-dopamine targets have been investigated to treat CUD. For example, Topiramate, an FDA approved medication for epilepsy and migraines that acts as a glutamate antagonist and γ-aminobutyric acid (GABA) agonist, increased abstinence in patients with CUD (Johnson et al., 2013). However, the effects were small and concerns regarding topiramate’s cognitive side effects mitigated interest in this medication (Rass et al., 2015). Disulfiram, an approved medication for alcohol use disorder has also been investigated for CUD and shown a small effect in improving abstinence (Traccis et al., 2024), but its high toxicity has limited further developments. Finally, bupropion, an FDA approved treatment for depression was also found to increase rates of abstinence among users, but its effects are small and findings inconsistent (Chen et al., 2020; Traccis et al., 2024).

More recently, pre-clinical studies on the impact of cocaine on the cerebrovasculature and neuronal calcium have revealed potential new treatments for CUD. These new potential therapies target channels associated with the pathophysiology of cocaine’s neurotoxicity and cocaine-induced cerebral ischemia. In a rat self-administration model, the L-type calcium channel blocker nifedipine blunted neuronal calcium accumulation and attenuated the reduction in tissue oxygenation following a cocaine challenge. Animals treated with nifedipine also displayed decreased cocaine intake and loss of cocaine reinstatement following extinction (Du et al., 2020; Du et al., 2021).

The efficacy of NMDA antagonist for treatment of CUD has also been investigated. Cocaine naïve rodents pretreated with memantine (NDMA antagonist) prior to a cocaine challenge showed a shortened duration of calcium influx with improved cerebral hemodynamics as compared to controls (Du et al., 2022). Taken together, these pre-clinical findings suggest that L-type calcium channels blockers and NMDA antagonists have potential as treatment to alleviate neuronal damage caused by increased calcium influx and chronic ischemia.

## 8 Conclusion

In this review, we sought to evaluate reductions in CBF as a potential mechanism in the relationship between cocaine and long-term neurological complications. In doing so, we explored the current work and understanding of how cocaine perturbations of the vascular system disrupt hemodynamic interactions with neuronal cells relevant to brain functional changes including changes in functional connectivity. Pre-clinical work has characterized the cerebral vascular response following acute cocaine and the adaptations to chronic cocaine exposures that are relevant for understanding the clinical findings of CBF and functional connectivity disruptions in CUD. Though significant advances have been made in delineating the effects of cocaine in the brain, further investigations are needed to better understand the pathophysiology of CUD including 1) characterization of the complex changes in neuronal and neurotransmitter activity that occurs with chronic use; 2) expanding the investigation on the roles of astrocytes in regulating the cerebral vasculature and neuronal activity; and 3) exploring the local microenvironment in the reactivity of neurons and astrocytes.

## Figures and Tables

**Table:** 

**Abbreviation**	Terminology
**CBF**	Cerebral Blood Flow
**CBFv**	Cerebral Blood Flow Velocity
**MCA**	Middle Cerebral Artery
**ACA**	Anterior Cerebral Artery
**PCA**	Posterior Cerebral Artery
**TCD**	Transcranial Doppler
**PI**	Pulsatility Index
**BBB**	Blood Brain Barrier
**PET**	Positron Emission Tomography
**μODT**	Ultrahigh Resolution Optical Imaging
**SPECT**	Single Photon Emission Tomography
**BOLD**	Blood Oxygen Level Dependent
**OISI**	Optical Intrinsic Signal Imaging
**OFI**	Optical Fluorescence Imaging
**fMRI**	Functional Magnetic Resonance Imaging
**FAIR**	Flow-sensitive Alternating Inversion Recovery
**LDF**	Laser Doppler Flowmetry
**EEG**	Electroencephalography
**LFO**	Low Frequency Oscillation
**LFP**	Local Field Potential
**FP**	Field Potential
**Ca** ^**2+**^	Calcium
**HbO**_**2**_	Oxygenated Hemoglobin
**HbR**	Deoxygenated Hemoglobin
**PFC**	Prefrontal Cortex
**mPFC**	Medical Prefrontal Cortex
**PrL**	Prelimbic Cortex
**OFC**	Orbitofrontal Cortex
**ACC**	Anterior Cingulate Cortex
**VTA**	Ventral Tegmental Area
**NAc**	Nucleus Accumbens
**D1r**	Dopamine-1 Receptors
**D2r**	Dopamine-2 Receptors
**MSN**	Medium Spiny Neurons
**DA**	Dopamine
**ATP**	Adenosine Triphosphate
**20-HETE**	20- hydroxyeicosatetraeonic acid
**iNOS**	Inducible Nitric Oxide Synthase
**NMDA**	N-methyl-D-aspartate
**LgA**	Long Access
**ShA**	Short Access
**IV**	Intravenous
**CUD**	Cocaine Use Disorder
**DREADD**	Designer Receptor Exclusively Activated by Designer drugs
**HAND**	HIV-Associated Neurocognitive Disorder
