# Supplementary material for: Neurovascular effects of cocaine: relevance to addiction
Source: Front Pharmacol. 2024 Feb 22;15:1357422. doi: 10.3389/fphar.2024.1357422 (PMC10917943; doi:10.3389/fphar.2024.1357422)
Supplement: Supplementary file 1 [file Table1.docx]

**Supplementary Table 1: CUD Treatment**

| **Author (year)** | **Study Type** | **Study Goal** | **Primary Findings** |
| --- | --- | --- | --- |
| Brandt et. al (2021) | Literature Review | Assess the current state of knowledge of available treatments for CUD | - Currently approved medications such as amphetamines, modafinils, and topiramate require further long-term studies. |
| Graboski et. al (2004) | Prospective Cohort | To determine the efficacy of combining methadone with either d-amphetamine or risperidone for the treatment of CUD | - Patients on 30/60mg d-amphetamine had reduced cocaine intake. - No change in cocaine use when risperidone was added to methadone. |
| Wanat et. al (2009) | Literature Review | Review current understanding of dopamine system and how drugs of abuse effect the rate of dopamine firing | - Cocaine and amphetamines alter dopaminergic function through multiple pathways including altering both tonic and phasic firing rates and increasing ratio of AMPA/ NMDA receptors. |
| Shearer et. al (2003) | Randomized Controlled Trial | Determine whether a placebo-controlled trial of dexamphetamine replacement for CUD can be conducted | - Percentage of cocaine-positive urines decreased. - Reduction in self-reported cocaine use and craving. |
| Volkow et. al (2009) | Prospective Cohort | Assess modafinil therapeutic effects on extracellular dopamine | - Modafinil effectively blocked dopamine transporters. |
| Sangroula et. al (2017) | Systematic Review and Meta-Analysis | Evaluate the safety and efficacy of modafinil for CUD | - 11 studies with 896 participants. - 6 studies concluded modafinil increased abstinence rates. |
| Sanna et. al (2019) | Prospective Cohort | Evaluate effectiveness of targeted PFC intermittent theta burst stimulation (iTBS) on cocaine craving and intake | - Both repetitive transcranial stimulation and iTBS lead to decreased cocaine craving and intake. |
| Terrano et. al (2016) | Randomized Controlled Trial | Impact of repetitive transcranial magnetic stimulation (rTMS) of the left dorsal lateral prefrontal cortex on CUD | - Treatment with rTMS was associated with an increase in cocaine free-urine drug tests |
| Johnson et. al (2013) | Randomized Controlled Trial | Evaluate topiramate for use to treat CUD | - Topiramate increased the number of days where cocaine was not used. - Topiramate decreased cocaine cravings. |
| Chan et. al (2019) | Systematic Review and Meta-Analysis | Determine effectiveness of pharmacotherapy for CUD | - Study reviewed multiple psychopharmacotherapy and their impact on cocaine abstinence, use, and relapse as well as reports on the strength of the evidence. |
| Du et. al (2021) | Pre-clinical | Determine the effect of Nifedipine, an L-type calcium channel blocker, on cerebral hemodynamics and neuronal activity following acute cocaine. | - Nifedipine reduced transient ischemia associated with acute cocaine use. - Cocaine induced neuronal calcium influx was attenuated with Nifedipine treatment. |
| Du et. al (2020) | Pre-clinical | Assess behavioral, hemodynamic, and neuronal changes associated with nifedipine in a cocaine self-administration rodent model | - Treatment with nifedipine reduced cocaine intake and blocked reinstatement in self-administration model. |
| Du et. al (2022) | Pre-clinical | Evaluate the impact of HIV-1 on cocaine-induced hemodynamic and neuronal changes as well as the impact of memantine on the pathology | - Pretreatment with memantine reduced cocaine-induced hemodynamic and neuronal changes. |
| Traccis et. al (2024) | Systematic Review | Asses the utility of disulfiram for CUD | - Disulfiram may improve abstinence among patients with CUD. |
